# A Community-Based Participatory Research Approach to Understand Urban Latino Parent's Oral Health Knowledge and Beliefs

**DOI:** 10.1155/2017/9418305

**Published:** 2017-03-29

**Authors:** Tamanna Tiwari, Nayanjot Rai, Eivi Colmenero, Hilda Gonzalez, Mirna Castro

**Affiliations:** ^1^School of Dental Medicine, University of Colorado Anschutz, Medical Campus, Aurora, CO, USA; ^2^Servicios de La Raza, Denver, CO, USA

## Abstract

The aim of this study is to describe oral health knowledge, behaviors, and beliefs of Latino parents with children under the ages of 6 years and to conduct a needs assessment with Latino families to better understand the challenges in maintaining oral health for their children. The investigator collaborated with a community serving the organization to recruit Latino primary caregivers for focus groups interviews and 30 primary caregivers were recruited. The focus groups data was transcribed and analyzed using a grounded theory approach using QDA Miner software. Findings from the focus groups demonstrate that the primary caregivers described barriers in maintaining oral health for their children including cultural barriers, child's temperament, lack of time, and easy access to high-risk foods. All participants said that they wanted to receive information on the oral health of their children; they wanted the dentist or the hygienist to demonstrate oral hygiene practices and explain to them the reasons for oral health behaviors. Although the primary caregivers recognized some factors related to caries development, their knowledge was limited in depth. Culturally appropriate oral health education is required for this population, which could lead to more adherent oral health behavior and a higher sense of self-efficacy in Latino parents.

## 1. Introduction

Parental oral health knowledge and behaviors are critical for prevention of early childhood caries (ECC) in young children [1]. Among Latino families in the US, children are more likely to experience ECC than non-Hispanic white and non-Hispanic black children [2]. Also, Latino children have the lowest average number of dental visits of all racial and ethnic groups and the highest rate of untreated tooth decay [2, 3]. According to a recent report based on the National Health and Nutrition Examination Survey, 2011-2012, dental caries prevalence was higher for Hispanic (46%) children than for non-Hispanic white children (31%) aged 2–8 [4]. Among Colorado's Latino population, oral health screenings conducted by the Colorado Department of Public Health and Environment in 2011-2012 revealed 55% of Latino children, aged 5-6 years, have had dental caries experience, while 32% of white children in the same age group had experienced dental caries [5].

Reasons for disparities in ECC prevalence in Latino children are not well studied. Initial research has shown that the Latino population experiences oral health disparities related to access to care [6]. It has been suggested that Latino mothers might lack knowledge related to risk factors associated with dental caries [7], and cultural beliefs and norms may negatively influence oral health behaviors [8]. Few studies have focused on the oral health knowledge, attitudes, and practices of Latino mothers/parents. Existing studies show that Latina mothers are aware of sugar consumption and inappropriate bottle use being the risk factors for dental caries, although the depth of knowledge was limited [7, 9, 10]. In a qualitative study, Latina mothers strongly agreed that oral hygiene routines contribute to good habits in children, but they were often inconsistent in adherence and had limited depth of knowledge about the benefits of fluoride [7]. Latino primary caregivers had limited knowledge about the etiology of dental caries and preventive concepts [9]. Another study found that Latino parent's beliefs about sugar-sweetened beverages were limited and many parents did not understand nutritional labeling related to store bought beverages; they viewed some beverages as healthy for their children despite high sugar content [11].

This purpose of this study was to conduct a needs assessment with Latino families to better understand the challenges in maintaining oral health for their children. The study followed community-based participatory research (CBPR) methodology to develop and conduct the study. CBPR is an approach that intends to involve community members, stakeholders, and community organizations representative, with academic investigators in all aspects of the research processes, and enhancing integration of the produced knowledge into the community [12].

Following the guidelines of CBPR methodology, goals for the academic-community partnership were developed and followed throughout the research conduction. We started the partnership development process before the grant application was submitted and had set the responsibility and expectations for both the academic and community team. The mission of the partnership and partner roles were decided before the grant application. The goals of this partnership were to develop a deeper understanding of the oral health knowledge, oral health behavior, and attitudes of Latino parents towards their children. The academic researcher relied on the community partner's expertise in connecting with the community and recruiting community members, mainly primary caregivers (mothers, grandmothers, and fathers) of preschool children for the focus groups. The community partners relied on the academic researcher to conduct the research and dissemination of the results.

## 2. Methods

The study was approved by the Colorado Multiple Institution Review Board. Thirty Latino primary caregivers who had at least one child under the age of six were recruited. The study enrolled the primary caregivers for the child, which will include grandparents and other relatives, apart from the mother and father of the child. Information about the study will be provided in English and Spanish, with a consent form detailing the approach of the study. All participants were recruited by Servicios Da La Raza's staff, which is a Latino-serving community organization working in the Denver Metro Area. The community organization approached Head Starts and kindergartens to recruit participants for the focus groups. Six focus groups were conducted. The focus group was conducted using a semi-structured focus group guide, consisting of ten open-ended questions that were developed by the investigators before the start of the study. The questions were developed based on previous qualitative studies conducted with Latino communities [7, 9, 11] and based on the experience of the investigator and the community partners working with the Latino population. A translator was provided for all the focus groups. Each session lasted 45–50 minutes and the time taken by the translator was additional.

All focus groups were digitally recorded, translated from Spanish, and transcribed into English. Grounded theory approach was used to develop the code structure. A systematic approach that allows for open discovery of emergent concepts with a focus on generating a theory was used to analyze the data. With this approach, the coding was done using a purely inductive technique, which helped to minimize the potential for “forcing” a preconceived result and thus provide for a more valid reflection of “the ground” or the true experiences of participants. Quotations provided in the results section reflect some sentiments and comments expressed by mothers during the focus groups. Both the research and a community partner independently coded the data and then conducted several meetings to discuss the codes, and once they felt saturation was reached they finalized the codebook.

The focus groups were analyzed using QDA Miner. The coding frequency was calculated for all codes under each of the three themes, using the frequency tool within the QDA Miner software. The code frequencies represent the percentage of the codes being repeated under each theme in the entire data.

## 3. Results

### 3.1. Focus Group Findings

Focus group participants were primary caregivers of children under the age of six years. There were about 20 mothers, seven grandmothers, one father, and two aunts who participated in the focus groups. The focus group themes and codes are reported in Box 1.

#### 3.1.1. Etiology of Dental Caries

**Figure 1:**
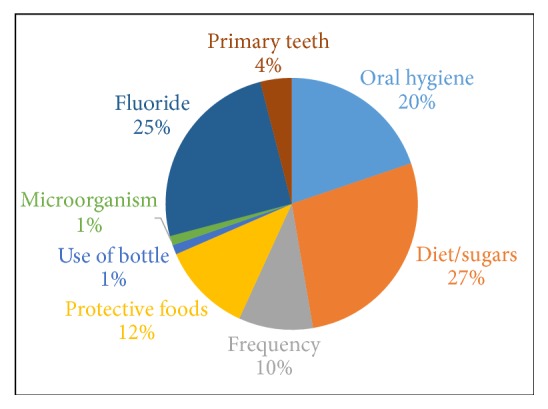
Percentage of codes that were repeated under etiology of dental caries.

Many factors were reported as being responsible for causing dental caries in children, including diet, poor oral hygiene, the frequency of consumption of sugary foods and drinks, and the role of microorganisms in development and progress of dental caries (Figure 1). Primary caregivers reported that diet was a major factor in dental caries development and gave examples of high sugar foods, such as candies, soda, cookies, and juice. About 28 percent of the participants reported some sugary food being responsible for the development of dental caries in their children (Figure 1). Participants mentioned the frequency of consumption of these sugary foods and drink as a cause of dental caries in their children. The frequency code appeared about 10 percent in the transcripts, within the theme for etiology of dental caries. Participants said that their children had soda or juice 2-3 times a day, but the frequency of consumption increased over the weekends or while visiting the family, where the participant had less “control” of the food choices of their children.

Participant comment: “*There is a lot of sugar in everything, things that you do not even know about. If they go to sleep at night without brushing their teeth, it just stays on their teeth all night*.”

Participant comment:* “I think the frequency is going to be the biggest factor. How may time they drink soda is important to manage?”*

Fruits and milk were mentioned as protective foods (12%) for teeth, as it has calcium, which is good for teeth. Milk was specified as protective food repeatedly by most parents, and they emphasized that it was good for teeth. Only two participants mentioned that milk should not be given to the child during bedtime and that adding sugar to milk increases the risk for caries development in children.

Participant comment: “I know that milk is good, it has calcium, but if you put your child to sleep with a bottle then that's going to decay their teeth, so you have to be careful.”

Only a handful of participants knew that microorganism caused caries in their children. The code appears less than 2 percent within the theme for etiology of dental caries (Figure 1). Even after several probes, participants did not mention microorganism as one of the causes for the development of dental caries. Most of the participants said that sugary foods were the main reason for caries development and had not heard that “bugs” were responsible for cavities. Most of the focus groups participants related poor oral hygiene practices to dental caries development. They understood the importance of good oral hygiene and 30 percent of the focus group thought teeth should be brushed at least two times daily. They also spoke about the importance of supervising their children while brushing or helping them brush their teeth. However, more than half of the participants said that they did not supervise their children over the age of 4-5 years during brushing. They said it was hard to find time to do that and thought they told their children to brush in the morning and at bedtime.

Another important discussion was based on fluoride and its role in dental caries prevention. Only two participants in the entire group knew the sources of fluoride including tap water, toothpaste, and fluoride varnish. Mechanism of action of fluoride in the prevention of caries was not known. Most participants had not heard about fluoride, and when probed they reported that neither the pediatrician nor the dentist had mentioned fluoride. They had no knowledge that tap water was the source of fluoride. Additionally, when probed about fluoride varnish, some of the participants remember their children receiving fluoride varnish during dentist appointments; they did not know that it had fluoride and recollected that it was mentioned as “vitamins for teeth.”

#### 3.1.2. Experience of Parents at Dental Visits

**Figure 2:**
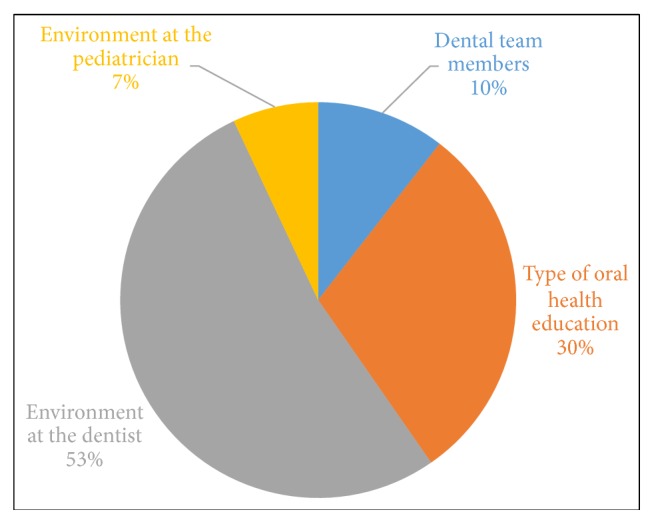
Percentage of codes that were repeated under the theme experience of parents under dental visits.

All the participants said that they have a dental home for their children or grandchildren. When discussing their experience during dental visits, most participants said that the environment at the dental clinic made a difference in changing their oral health behaviors. The code “environment at the dentist” appeared 53 percent within the theme (Figure 2). Two types of environments were described. Few participants received a supportive environment, where the dental team gave preventive recommendations to the children; they had toys, information pamphlet, and videos in the waiting area that captured the parent and child's attention.

Participant comment:* “At the clinic where I go, in the waiting room, they show some videos. A video got my attention. My children also paid attention to it because it shows you a soda and asks you, how many tablespoons of sugar does a soda have? - 12 tablespoons sugar. They say, in your right mind, would you give 12 tablespoons of sugar to your child?”*

However, the majority of the participants were not satisfied with their dentist. Interactions were more with the hygienist, and dental assistants and dentist spent the least time interacting and educating the parents.

Participant comment:* “I feel like dentists have not been very personable. I think it is always very fast pace. You spend most time maybe with a technician rather than with the dentist, and they don't provide enough information to really understand how to prevent cavities.”*

Few parents reported that some intricacies related to treatment decision were not discussed in detail and other stated that reasons for applying fluoride varnish at the end of the visit were not explained. Nevertheless, for some parents, the environment at the dentist was a cause of frustration because they felt judged. These parents had children who had higher dental caries experience and went more often to the dental clinic.

Participant comment:* “I have these frustrating conversations with the dentist and they almost think that you are lying. I am like, No, I am not lying, and they do not drink sugary drinks. He was off the bottle very early. I have tried to make it different from my older son, and it still happened the same way.”*

Apart from the dental office, the participants also received some oral health knowledge from the pediatrician or at Women Infant and Child Clinics. The participants wanted the dentist to demonstrate the oral hygiene practices or provide the reason for a recommended oral health behavior. Participants mentioned that some dental offices had a show and tell method that was very useful in engaging and educating the children. Participants felt motivated when the dentist or other medical personnel were polite and showed concern for their children's oral health, rather than just giving them instructions.

#### 3.1.3. Oral Health Beliefs of Parents

**Figure 3:**
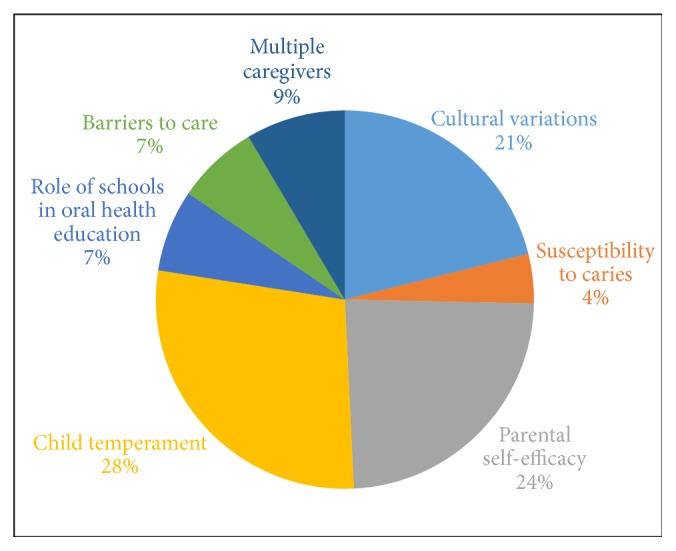
Percentage of codes that were repeated under the theme oral health beliefs of parents.

Child temperament had the highest frequency (28 percent) with this theme (Figure 3). Participants faced challenges when brushing their children's teeth, mainly those related to the child's behavior, and most mothers believed this affected their children's oral health. They were not able to engage their children in brushing when they started to cry or refused to brush. In addition, they said it was difficult to make it into an interesting exercise for the child. Child temperament was also mentioned in restricting them from consuming sugary foods and drinks. If the child had a liking for soda or candies, the parents struggled in controlling the frequency of consumption.

Participant comment:* “We prohibit all sweets, but that is what she likes most – sweets, soda, apple soda, apples. What can I do?”*

Lack of time on their part was another challenge. Participants mentioned jobs and other chores that prevented them from finding time to supervise their children while brushing or encouraging their children to brush. Self-efficacy of the participants was an important factor controlling their oral health behaviors towards their children. Parents thought they had no control over their child's food habits and they felt helpless.

Participant comment:* “If they are hungry and they just grab something; it is hard to say no.”*

Participants discussed cultural variations in their community related to oral health knowledge and access to care. Some participants mentioned they struggled to overcome peer and familial pressure when they took their children to the dentist for preventive visits. They mentioned their family and friends suggested visiting the dentist if the child has pain and that preventive dentist's visits were unnecessary. Another topic they discussed was about multiple caregivers, especially grandmothers and aunts who provided care when the mother was at work. Participants had little or no control over the feeding practices of additional caregivers. They mentioned their children have easy access to high-risk foods such as soda and candies in the US as compared to children in their (home) countries, which they deemed as a negative effect on the oral health of their children.

Participant comment:* “My mother-in-law lives in Mexico. When she comes, she stays for two months, and when she is watching him, it is a harder to control his diet, because she eats breakfast with Coca-Cola. So then trying to get her not to give the baby these juices and stuff gets a little harder.”*

The role of schools and Head Starts in educating the children about oral health was emphasized by many participants. Children spend most of the time at schools that would be a suitable place for the child to learn about oral health.

## 4. Discussion

The results of this study highlight that although Latino parents have some understanding of factors associated with dental caries their knowledge did not translate into positive oral health behaviors, however. The focus group findings have provided some in-depth information on the challenges these parents may face in implementing their knowledge into actions. Few culturally tailored studies conducted with Latino parents to improve oral health knowledge and behaviors have been well accepted by the community and have shown sustained improvements in oral health knowledge and behavior change in Latino parents [6, 13].

Child temperament and self-efficacy of the parents were two concepts that surfaced several times during the discussions. Parents discussed their child's resistance towards tooth brushing and lack of time in supervising the children during brushing as some other reasons for poor oral hygiene for the children. Evidence suggests child temperament has been strongly associated with ECC. Positive temperament appears (such as positive anticipation and soothability) protective, and negative temperament (such as attentional shifting, fear, frustration, low-intensity pleasure, and sadness) may increase the risk of ECC [14, 15]. It also has been shown that negative child temperaments can exacerbate parental stress and worsen parental feeding and oral health practices [15].

Additionally, some of the participants reported the lack of self-confidence in engaging the child in oral hygiene activities mostly because they did not know how to teach the oral hygiene techniques to their child. The dentist, physician, and the schools were held responsible for teaching oral hygiene to children, and only a few parents felt that they were accountable for their child oral health. Skill building exercises with parents have shown improvements in tooth brushing behaviors in parents for their children [13, 16]. A peer based skill building study concluded that, after a 3-month intervention of teaching tooth brushing skills to parents, there was a significant increase in parents' confidence to ensure brushing twice a day, as well as in perceptions of the importance of tooth brushing, and in self-efficacy for tooth brushing skills [16].

In addition to providing oral health knowledge, approaches such as Motivational Interviewing (MI), which provides supportive guidance for choosing behavioral goals and strategies, may be more appropriate and culturally sensitive in a Latino population [17]. Few studies that have used MI have reported that this approach can positively impact behaviors addressing infant feeding practices and diet, oral hygiene behaviors, and dental attendance [18].

Limitations of this study include the relatively small sample size and a convenience sample. These results may not be generalizable. Further research is warranted with a larger sample to validate these results.

## Figures and Tables

**Box 1:**
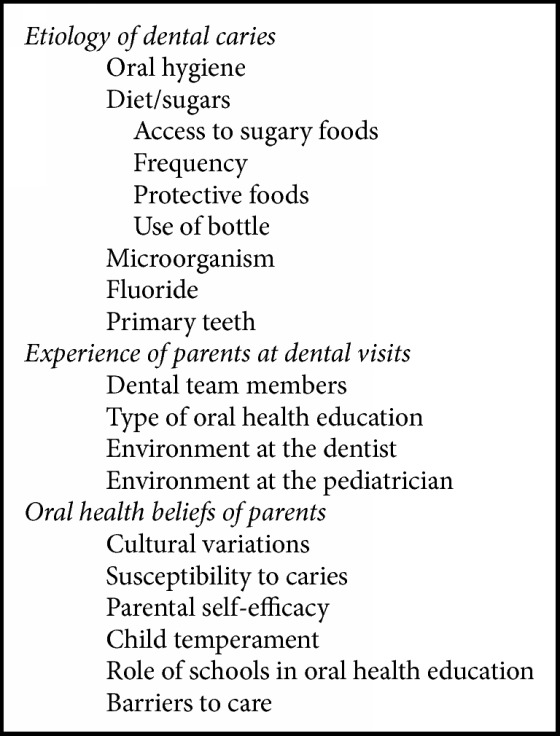
Themes and codes.
